# A Cross-Sectional Study of the Prevalence of and Risk Factors for Suicidal Ideation Among the Elderly in Nursing Homes in Hunan Province, China

**DOI:** 10.3389/fpsyt.2020.00339

**Published:** 2020-04-30

**Authors:** Yu Nie, Zhao Hu, Tingting Zhu, Huilan Xu

**Affiliations:** ^1^Department of Social Medicine and Health Management, Xiangya School of Public Health, Central South University, Changsha, China; ^2^Department of Scientific Research Management, Shanghai Health Development Research Center, Shanghai, China

**Keywords:** suicidal ideation, elderly, interaction, nursing home, risk factors

## Abstract

Our study aims to explore the risk factors for suicidal ideation and their interaction among the elderly in nursing homes in Hunan province, China. A cross-sectional study was conducted among the elderly in nursing homes in Hunan Province. Twenty-four nursing homes were selected by multistage cluster random sampling, and 817 elderly residents were investigated using a set of structured questionnaires. The main outcome measures included general information, suicidal ideation, depression symptoms, social support, activities of daily living (ADL), stressful life events, and sleep quality. Multivariate binary logistic regression was performed to explore the risk factors for suicidal ideation among the elderly in nursing homes, and additive interaction was used to analyze the interaction between risk factors. The prevalence of suicidal ideation among the elderly in nursing homes in Hunan province was 17.9% (95% confidence interval(CI): 15.2%, 20.6%). Living in a rural area (odds ratio(OR)=1.88, 95% CI: 1.03, 3.44), infrequent visits from relatives (OR=2.61, 95% CI: 1.42, 4.78), history of chronic disease (OR=2.34, 95% CI: 1.09, 5.01), depression symptoms (OR=8.11, 95% CI: 4.52, 14.54), lower social support (OR=3.85, 95% CI: 1.94, 7.61), and ADL disability status (OR=4.38, 95% CI: 2.10, 9.14) increased the risk of suicidal ideation. Additive interactions were detected between depression symptoms and ADL status, with a relative excess risk due to interaction (RERI) of 8.73 (95% CI: 2.04, 15.43), and between depression symptoms and social support, with an RERI of 5.98 (95% CI: 0.86, 11.10). The prevalence of suicidal ideation among the elderly in nursing homes is relatively high. Both physical conditions and psychosocial factors were associated with suicidal ideation among the elderly. These findings have significant implications for the prediction and prevention of suicidal behaviors.

## Introduction

Suicide has gradually become a major public health problem; it is ranked the 13th leading cause of death and accounts for 1.4% of all deaths (1). In China, suicide is the 5th leading cause of death, and the incidence of suicide is estimated to be 6.6/100,000 people per year (2). Moreover, China has the third highest rate of suicide among the elderly worldwide, and adults over 65 years of age had the highest rate of completed suicide: 44.3 to 200 per 100,000 persons (3). However, suicide rates among the elderly have either declined or remained unchanged in the past decades (4).

Suicidal ideation is the early phase of suicide and is defined as active or passive thoughts about killing oneself at some point or phase in life (5, 6). Moreover, suicidal ideation is one of the strongest predictors of suicidal behavior and predicts an increased risk of eventual death by suicide (7). Globally, the lifetime prevalence of suicidal ideation is approximately 9.2% (8). In China, according to a meta-analysis in 2005, the estimated lifetime prevalence of suicidal ideation was 3.9% (95% confidence interval (CI): 2.5%, 6.0%) in the general population (9). Another meta-analysis conducted in 2014 that included 11,526 subjects indicated that the prevalence of suicidal ideation among Chinese elderly adults ranged from 2.2% to 21.5%. The pooled prevalence was 12.6% in mainland China (10). Therefore, with the ageing of the population in China, more attention should be paid to the pattern of suicidal ideation considering its relationship with suicidal behavior.

As mentioned above, ageing is currently an increasing problem in China, and the population of elderly individuals is increasing rapidly and accounting for an increasingly large proportion of the general population. According to the data of the Sixth National Population Census in 2010, the number of people aged 60 years and older was approximately 177 million, accounting for 13.26% of the total population. The U.N. Commission on Population Development predicted that the elderly population aged 60 years and older in China will reach 243.8 million by 2020, accounting for 17.1% of the total population. However, the reduction in family size due to China’s previous one-child policy has led to a shortage of family caregivers who can provide home-based long-term care for older adults. Specifically, the current pattern of “421/422” families (four grandparents, two parents, and one or two children) creates a major problem for informal family caregivers (11). All of these factors have increased the demands and requirements for nursing homes (12). According to data from the Ministry of Civil Affairs of the People’s Republic of China, the number of nursing homes in China has grown from 5.1 thousand in 2015 to 13.9 thousand in 2017. However, many studies have indicated that elderly people living in nursing homes have a greater likelihood of suffering from nutritional deficiencies and poor psychological health and have a higher incidence of suicidal ideation compared to community-dwelling older adults (13, 14). As the elderly population grows, the severe physical and mental health problems pose a considerable challenge to nursing homes and health care systems.

It is of great significance for suicide prevention to identify individuals with suicidal ideation and intervene to reduce the occurrence of suicidal behaviors. Several studies in the current literature have demonstrated that many factors are associated with suicidal ideation among older adults, such as marital status (15), social support (16), economic status (17), sleep quality (18), depression, anxiety, and loneliness (14, 16, 19). However, most of those studies were conducted among community-dwelling older adults. Therefore, our study aimed to investigate the prevalence of suicidal ideation among the elderly in nursing homes in Hunan province, China. Moreover, we intended to explore the risk factors for suicidal thought and their interactions. We hope that this study will provide some valuable information for suicide prevention among the elderly in nursing homes in China.

## Materials and Methods

### Study Design

This cross-sectional study was conducted in Changsha, Hengyang, and Yiyang City in Hunan Province, China, from October to December 2018. The study was approved by Xiangya School of Public Health at Central South University (No.XYGW-2018-49). Written informed consent was obtained from all participants of this study.

### Sample Size Calculation

Sample size was calculated using the formula for cross-sectional studies, as follows:

$$N=\frac{Z_{1-\alpha /2}^{2}p\left(1-p\right)}{d^{2}}$$

where *Z*_1-α/2_ = 1.96 when α=0.05, *p* is the prevalence of suicidal ideation [which was 20% according to a previous study (14)], and *d* is admissible error (which was 3% here). According to the formula, the estimated sample size was 751, which included an extra 10% to allow for subjects lost during the study.

### Study Population and Procedure

A multi-staged cluster randomized sampling method was used to select a representative sample of elderly adults living in nursing homes in Hunan Province. In the first stage, one city each from northern Hunan, southern Hunan, and central Hunan (Changsha City, Hengyang City, and Yiyang City) was selected based on geographical regions. One county was then randomly chosen from each selected city: Changsha County, Hengyang County, and Yuanjiang County. In the second stage, two urban districts (Kaifu and Yuelu) from Changsha City and two townships (Xingsha and Tiaoma) from Changsha county were randomly selected. Similarly, Yanfeng and Shigu from Hengyang City and Xidu and Jingtou from Hengyang County were randomly selected. Ziyang and Heshan from Yiyang City and Qionghu and Caowei from Yuanjiang County were randomly chosen. In the third stage, two nursing homes were randomly selected from each selected urban district and township; a total of 24 nursing homes were finally selected.

Resident populations in selected nursing homes were included in our study if they met the following inclusion criteria: (1) age 60 years and above, (2) duration since entering the nursing home of more than one year, (3) physical and mental ability to participate in interviews. Participants were excluded if they had: (1) a severe hearing impairment or a language barrier, (2) a history of severe cognitive deficits diagnosed by a physician, or (3) a terminal illness.

**Figure 1:**
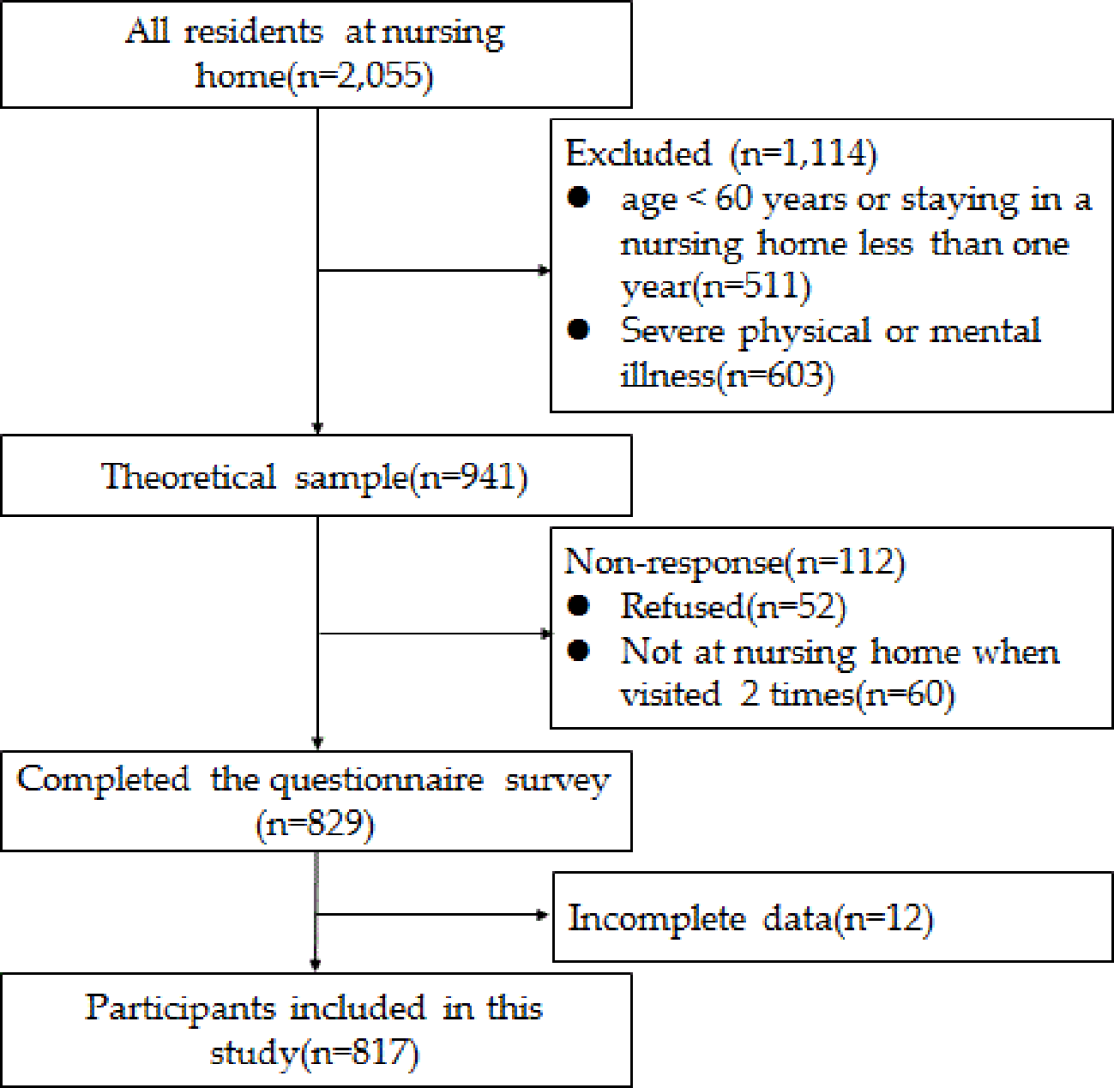
Response of Subjects enrollment flowchart.

There were 2,055 residents in the 24 nursing homes; 511 were excluded because they were younger than 60 years of age or had been in a nursing home less than one year, and 603 were excluded due to severe physical or mental illness. The theoretical sample size was 941 people, of which 112 were not investigated for various reasons (response rate: 88.1%). Of the remaining 829 elderly adults, 12 were excluded due to incomplete data. Finally, a total of 817 elderly adults were included in the data analysis in this study. The enrolment procedure is shown in **Figure 1**.

### Data Collection and Measurements

Socio-demographic information was collected, including residence before entering into nursing home, gender, age, education level, marital status, monthly personal income, medical insurance, duration of residence in the nursing home, number of children, smoking status, alcohol consumption status, history of falls in the past year, history of chronic disease, and the visitation frequency of relatives. Marital status was dichotomized into stable and unstable. Unstable marital status included divorce, loss of a partner, and never married. Education level was classified into three categories: primary school or below, junior high school, and senior high school and above. Smoking was defined as an average of at least one cigarette per day in the last year. Alcohol consumption was defined as drinking a glass of wine per day in the last week. For the purpose of this study, chronic disease referred to chronic non-communicable diseases, including cardiovascular disease, hypertension, chronic obstructive pulmonary disease, type 2 diabetes, and others. The frequency of visits from relatives was assessed through a single item that asked the participants to select the frequency of visits from their relatives from the following choices: once or more per week, 1-3 times per month, and less than once per month. The answers were used to classify the participants into two groups: high (once or more per month) and low (less than once per month).

Suicidal ideation was determined using the Chinese Version of the Beck Scale. The scale assesses the intensity of suicidal attitudes and suicidal tendency in the past week as well as the periods of greatest depression, and has been proven to have good validity and reliability in China (20). Participants were identified as having suicidal ideation when they gave positive answers for item 4 (How willing are you to commit suicide)? or item 5 (To what extent would you end your life passively)?.

Depressive symptoms were assessed by the Geriatric Depression Scale-30 (GDS-30), which has been widely used for the elderly worldwide (21). The validity and reliability of the GDS-30 have been extensively assessed in China (22, 23). The GDS consists of 30 true/false questions. The total score ranges from 0 to 30 points, and participants who score 11 and above are considered to have depression symptoms (24).

Social support among the elderly was assessed using the Social Support Rating Scale (SSRS), which has been widely used in China and has a Cronbach’s α=0.762 (25). The scale consists of three parts: objective support, subjective support, and availability of social support. In this study, subjects with lower social support were identified when the scale score was lower than the mean.

The Lawton and Brody activities of daily living (ADL) scale was used to assess the ADL status of the elderly adults in nursing homes (26). It consists of a physical self-maintenance scale and an instrumental ADL scale. Each item was scored from 1 to 4, and ADL disability status was determined when participants had a total score higher than 14 points.

Sleep quality in the past month among the elderly was assessed using the Pittsburgh Sleep Quality Index (PSQI) (27). It contains 18 items grouped into seven components and scored from 0 to 3, with 0=“not difficult” and 3= “very difficult”. The total score ranges from 0 to 21 points, and participants with a score of 5 and above are defined as having poor sleep quality.

Stressful life events were assessed using the life events scale for the elderly (LESE). The LESE was developed based on previous scales and has been proven to have reliability and validity among the Chinese elderly population (28). It includes 46 items grouped into three main domains: health-related problems, marital and family problems, and social and other problems. Participants are determined to have stressful life events when any of these events have occurred and they indicate that they feel stressed.

### Statistical Analysis

The data were analyzed using Statistic Package for Social Science (SPSS)version 18.0 (SPSS/IBM, Armonk, New York, USA) and Statistical Analysis System (SAS) version 9.2 (SAS Institute, Inc., Cary, NC, USA). Data are described as the mean ± standard deviation (SD) for continuous variables and n(%) for categorical variables. The stepwise Wald test binary logistic regression was used to identify risk factors for suicidal ideation. The strength of association was estimated as odds ratios (OR) and 95% confidence intervals (CI). The interaction between risk factors was calculated by the Excel table introduced by Andersson et al. (29). Three main indicators were used to determine the existence of additive interactions: the relative excess risk due to interaction (RERI), the attributable proportion due to interaction (AP), and the synergy index (S). There is no significant additive interaction when the RERI and AP are equal to 0 or S is equal to 1. All statistical tests were two-tailed, and *p* < 0.05 was considered statistically significant.

## Results

### Characteristics of the Study Subjects

Among the 817 participants, the average age was 79.10 ± 8.71 years, and 46% were male. Approximately 90% of the subjects had medical insurance and had at least one child. More than half of the participants had a monthly income lower than 3,000 CNY and had resided in a nursing home for less than 3 years. A total of 27.9% of them had been admitted in the past year. More than half of the individuals were visited once or more per month by relatives. A minority of the participants smoked and consumed alcohol and had depression symptoms. A majority of the individuals had experienced at least one type of chronic disease, had experienced at least one stressful life event, and had poor sleep quality. The results are shown in **Table 1**.

**Table 1:** Characteristics of the study subjects residing in nursing homes.

Variables	n	*%*
**Residence**		
Urban area	594	72.7
Rural area	223	27.3
**Gender**		
Male	376	46.0
Female	441	54.0
**Age**		
≤70 years	165	20.2
>70 years	652	79.8
**Education**		
Primary school and below	364	44.6
Junior high school	203	24.8
Senior high school and above	250	30.6
**Medical insurance**		
Yes	766	93.8
No	51	6.2
**Have one child or more**		
Yes	746	91.3
No	71	8.7
**Marital status**		
Stable	302	37.0
Unstable*	515	63.0
**Monthly personal income**		
≤3,000 CNY	568	69.5
>3,000 CNY	249	30.5
**Duration of admission**		
1-3 years	566	69.3
>3 years	251	30.7
**Frequency of visits from relatives**		
High	596	72.9
Low	221	27.1
**History of chronic disease**		
Yes	620	75.9
No	197	24.1
**Smoking**		
Yes	132	16.2
No	685	83.8
**Alcohol drinking**		
Yes	96	11.8
No	721	88.2
**History of fall**		
Yes	228	27.9
No	589	72.1
**Depression symptoms**		
Yes	294	36.0
No	523	64.0
**Social support**		
High (>30)	382	46.8
Low (≤30)	435	53.2
**Stressful life events**		
Yes	725	88.7
No	92	11.3
**ADL status**		
Normal	268	32.8
Disabled	549	67.2
**Sleep quality**		
Good	267	32.7
Poor	550	67.3

ADL, activities of daily living.

*including being widowed, divorced and never married.

### Prevalence of Suicidal Ideation

A total of 146 elderly adults (66 male, 80 female) living in a nursing home reported suicidal ideation in the past week. The prevalence was 17.9% (95% CI: 15.2%, 20.4%). Among them, 129 elderly adults reported having active suicidal ideation, for a prevalence of 15.8% (11.0% reported mild active suicidal ideation and 4.8% reported strong active suicidal ideation). A total of 143 elderly adults had passive suicidal ideation, for a prevalence of 17.5% (8.8% reported mild passive suicidal ideation and 8.7% supported strong passive suicidal ideation).

### Risk Factors Associated With Suicidal Ideation

Multivariate binary logistic regression analysis indicated that the risk factors for suicidal ideation among elderly adults living in nursing homes were living in a rural area (OR=1.88, 95% CI: 1.03, 3.44), infrequent visits from relatives (OR=2.61, 95% CI: 1.42, 4.78), history of chronic disease (OR=2.34, 95% CI: 1.09, 5.01), depression symptoms (OR=8.11, 95% CI: 4.52, 14.54), reduced social support (OR=3.85, 95% CI: 1.94, 7.61) and ADL disability (OR=4.38, 95% CI: 2.10, 9.14) after adjusting for variables such as age, gender, education, medical insurance, marital status, monthly income, duration of nursing home residency, having children, smoking, alcohol consumption, negative life events, history of falls, and sleep quality. The results are shown in **Table 2**.

**Table 2:** Factors associated with suicidal ideation among elderly adults living in nursing homes.

Variables	Participants(n)	Prevalence of SI (%)	Crude OR (95% CI)	Adjusted OR (95% CI)^†^
**Residence**				
Urban area	594	10.6	1.00	1.00
Rural area	223	37.2	5.00(3.43,7.28)	1.88(1.03,3.44)
**Frequency of visits from relatives**				
High	596	10.1	1.00	1.00
Low	221	38.9	5.69(3.89,8.32)	2.61(1.42,4.78)
**History of chronic disease**				
No	197	7.1	1.00	1.00
Yes	620	21.3	3.54(1.99,6.29)	2.34(1.09,5.01)
**Depression symptoms**				
No	523	3.6	1.00	1.00
Yes	294	43.2	20.17(12.08,33.69)	8.11(4.52,14.54)
**Social support**				
High	382	3.7	1.00	1.00
Low	435	30.3	11.45(6.47,20.28)	3.85(1.94,7.61)
**ADL status**				
Normal	268	4.9	1.00	1.00
Disabled	549	24.2	6.27(3.48,11.32)	4.38(2.10,9.14)

^†^Adjusted for age, gender, education, medical insurance, marital status, monthly income, duration of admission, having children, smoking, alcohol consumption, negative life events, history of falls, sleep quality, and all variables shown in the table.

SI, suicidal ideation; OR, odds ratio; CI, confidence interval; ADL, activities of daily living.

### Interaction of Risk Factors for Suicidal Ideation

In this study, additive interactions among risk factors for suicidal ideation were calculated. The regression coefficients and covariance matrix were generated by multivariate binary logistic regression after adjustment for all covariates, except for two factors whose interaction was being examined. The Excel table prepared by Andersson et al. was used to calculate RERI, AP, and S based on the results of the logistic regression.

Additive interactions were detected between depression symptoms and ADL status, with an RERI of 8.73 (95% CI: 2.04, 15.43) and an AP of 0.49 (95% CI: 0.28, 0.70) and between depression symptoms and social support, with an RERI of 5.98 (95% CI: 0.8, 11.10), an AP of 0.42 (95% CI: 0.17, 0.67), and an S of 1.83 (95% CI: 1.13, 2.98). No significant interaction was detected between other risk factors. The results are shown in **Table 3**.

**Table 3:** Interaction of risk factors for suicidal ideation.

Factor 1	Factor 2	Adjusted OR^†^	RERI	AP	S
Depression symptoms	ADL status		8.73(2.04,15.43)	0.49(0.28,0.70)	2.35(1.62,3.49)
−	−	1.00			
+	−	7.06(2.77,13.24)			
−	+	3.15(1.25,7.90)			
+	+	17.94(7.34,33.86)			
Depression symptoms	Social support		5.98(0.86,11.10)	0.42(0.17,0.67)	1.83(1.13,2.98)
−	−	1.00			
+	−	6.37(2.48,16.35)			
−	+	2.86(1.07,7.57)			
+	+	14.21(5.89,34.29)			

^†^Adjusted for age, gender, marital status, education, medical insurance, monthly income, duration of residency, having children, smoking, alcohol consumption, negative life events, history of falls, sleep quality, history of chronic disease, frequency of visits from relatives, and all variables shown in the table.

OR, odds ratio; RERI, the relative excess risk due to interaction; AP, the attributable proportion due to interaction; S, the synergy index; ADL, activities of daily living.

## Discussion

The present study selected 24 nursing homes in both urban and rural areas of Hunan province using a multistage cluster random sampling method. Based on a large population, our study found that the prevalence of suicidal ideation was 17.9% among elderly nursing home residents. This result is comparable to a previous study conducted among rural elderly Chinese adults living in nursing homes, which reported that 19.5% of participants had experienced suicidal ideation (14) in the past week. However, with similar institutions and populations, Malfent et al. estimated that the lifetime, one-year, and one-month prevalence of suicidal ideation among long-term care (LTC) residents was 35%, 11%, and 7% (30), respectively. Scocco et al. (31) reported that 30.8% of elderly nursing home residents admitted to having had thoughts of death or suicide during the previous month. Haight et al. reported that 12% of elderly residents who were newly relocated to LTC facilities had suicidal thoughts (32). An explanation for the variance in these estimates may be the ethnic and sociocultural differences between different countries. Moreover, this variance may result from differences in facilities, equipment, and quality of nurses and care among different institutions.

Even so, the prevalence of suicidal ideation among elderly nursing home residents seemed to be higher than that of older adults living in the community in both urban and rural areas. A study including 9,416 urban residents and 9,267 rural residents aged 60 years or older in China indicated that the prevalence of suicidal ideation in the previous month was 1.95% among urban residents and 3.60% among rural residents (33). One possible explanation is that elderly adults living in institutions are relatively older and have worse physical health and more chronic diseases compared to community-dwelling older adults (34, 35). Another possible explanation is that institutionalized elderly adults have lost some obscure protective factors that are available to community-dwelling adults, thus making them more vulnerable to suicidal ideation and suicide. Similar to a 15-year follow-up study conducted among American adults aged 60 years and older, the number of suicides among community-dwelling adults showed a downward trend, while the number of suicides in older adults in LTC showed no change (36).

Our study found that the prevalence of suicidal ideation among the institutionalized elderly who live in rural areas is higher than that of institutionalized elderly adults living in urban areas, which is consistent with other studies of community-dwelling elderly adults (37, 38). On the one hand, low socioeconomic status (SES) is more prevalent in rural areas than in urban areas and leads to poor utilization of health services. In addition, financial strain has been proven to be an independent risk factor for suicidal ideation (39). On the other hand, the equipment and personnel allocation in nursing homes in rural areas are inferior to those in urban regions, which leads to dissatisfaction with their ability to meet demands and causes a series of problems.

One interesting finding is that the frequency of visits from relatives is associated with the development of suicidal thoughts among elderly adults in nursing homes. Similar results were obtained in another study in China; that study demonstrated that frequent visits from children have a direct impact on loneliness and an indirect effect on suicidal thoughts using a path analysis method (14). In addition, the low frequency of visits from children represented a lack of filial piety to some extent, which is recognized as a major risk factor for suicidal ideation according to Chen YJ et al. (40). The association may be related to the need for emotional support among the elderly. Moreover, home visits from commissioned welfare volunteers were determined to be a protective factor against suicidal ideation among the elderly in Japan (41).

The present study also found that a history of chronic disease was associated with suicidal thoughts, which was consistent with other studies carried out in China and in other countries (42, 43). A study conducted among 5,514 rural elderly individuals in Shandong province, China, found that chronic disease had both direct and indirect impacts on suicidal ideation (44). Moreover, Handley et al. (45) found that within high psychological distress, lower physical functioning significantly increased the likelihood of suicidal ideation, with high distress and low functioning being associated with ideation in 50% of cases through a decision tree analysis. However, our study did not find significant interactions between chronic disease and other risk factors, and thus, further studies are needed.

Depression has been regarded as a main risk factor for suicidal ideation (14, 46). Our study found that institutionalized elderly adults with depression symptoms had an increased risk of suicidal ideation after adjusting for covariates (OR 8.11; 95% CI 4.52,14.54). The result was in accordance with other studies conducted both in the community and in care homes (17, 30). Moreover, studies also showed that depression was a very common mental health problem among the institutionalized elderly, and the GDS scale scores of elderly adults living in a care facility was higher than that of elderly adults living in the community (47). Based on this finding, depression screening and psychological interventions for institutionalized older people can be implemented to detect and protect vulnerable individuals.

Lack of social support is also a main risk factor for suicidal ideation, which is in agreement with the results of previous studies (48, 49). For instance, Raue et al. reported that subjective social support was independently associated with the presence of passive and active suicidal ideation among 539 elderly homecare patients (49). Social support may play a vital role in the Chinese population, whose value system emphasizes a collectivistic culture and interpersonal harmony (50). Furthermore, many studies have stated that social support moderates the relationship between other factors and suicidal thoughts (51). In our study, we found that social support has an interaction with depression symptoms. On the one hand, lower perceived social support may increase the risk of persistence of depression symptoms (52), thus increasing the risk of suicidal ideation. On the other hand, many studies have found that social support had a significant indirect effect on suicidal ideation *via* depression (53, 54). For instance, a study conducted among older Korean adults with hypertension found that social support mediated the relationship between depression and suicidal ideation (55). These results may explain the interaction between depression and social support to some degree.

ADL was also an important influencing factor for suicidal ideation; the institutionalized elderly with ADL disability status seemed to have a higher prevalence of suicidal ideation than those with normal ADL. The study conducted by Wei JW et al. (33) supports this finding. It may be that the Chinese elderly with ADL disability experienced feelings of low self-worth from feeling that they could not do anything and even caused trouble for their children. Moreover, our study found that ADL status is not only a risk factor for suicidal ideation but also has an additive interaction with depression. Although the underlying mechanism is unclear, we must realize that some people who have both ADL disability and depression symptoms have a higher rate of suicidal ideation. A study of 15,890 middle-aged and older Chinese adults found that ADL disability might have the potential to increase the risk of depression symptoms (56). Moreover, a study demonstrated a temporal and reciprocal relationship between ADL disability and depressive symptoms in later life, indicating that disability and depressive symptoms are mutually reinforcing over time (57). This mutual association may partly explain the presence of the interaction between MDD and ADL in cases of suicidal ideation.

In the current literature, this is the first study to explore the risk factors for suicidal ideation and their interactions among the elderly in nursing homes in China. The findings provided valuable information for suicidal prevention among elderly for such institutions. However, our study also has several limitations to address. First, temporal and causal relationships could not be assessed due to the cross-sectional study design. Second, there may be limitations in measurement accuracy and the classification of suicidal ideation based on partially retrospective self-reports. Third, a bias may be introduced to our results as participants with previous mental disorders or mild cognitive impairment were not controlled for. Finally, from the participants’ perspectives, there could be a recall bias of the condition as the subjects were asked for some events in the past month prior to study. Therefore, further studies are needed to confirm the findings and garner a deeper understanding.

## Data Availability Statement

The raw data supporting the conclusions of this article will be made available by the authors, without undue reservation, to any qualified researcher.

## Ethics Statement

The study was approved by Xiangya School of Public Health at Central South University (No.XYGW-2018-49). Written informed consent was obtained from all participants of this study.

## Author Contributions

HX developed the study. YN and TZ collected the data. YN and ZH conducted data analysis and wrote the manuscript. HX reviewed and revised the manuscript. All authors read and approved the final manuscript. YN and ZH contributed equally.

## Conflict of Interest

The authors declare that the research was conducted in the absence of any commercial or financial relationships that could be construed as a potential conflict of interest.
